# Prevalence and factors associated with HIV testing among young women in Ghana

**DOI:** 10.1186/s12879-024-09068-8

**Published:** 2024-04-19

**Authors:** Mainprice Akuoko Essuman, Hidaya Mohammed, Martha Suntah Kebir, Comfort Obiribea, Bright Opoku Ahinkorah

**Affiliations:** 1 Department of Medical Laboratory Science, School of Allied Health Sciences, College of Health and Allied Sciences, University of Cape Coast, Cape Coast, Ghana; 2 Department of Biological Sciences, Southern Illinois University Edwardsville, Edwardsville, IL USA; 3 Department of Microbiology and Immunology, School of Medical Sciences, College of Health and Allied Sciences, University of Cape Coast, Cape Coast, Ghana; 4 School of Clinical Medicine, University of New South Wales, Sydney, Australia; 5 REMS Consultancy Services, Sekondi-Takoradi, Western Region Ghana

**Keywords:** HIV, HIV testing, HIV prevention, Young women, Ghana

## Abstract

**Background:**

HIV/AIDS is a global health challenge and continues to threaten lives in sub-Saharan African countries such as Ghana. One of the important interventions for controlling its transmission is through testing and receiving medication. In this study, we present findings on the prevalence and factors associated with HIV testing among young women in Ghana.

**Methods:**

We used data from the 2014 Ghana Demographic and Health Survey comprising young women aged 15–24 years. We calculated the proportion of these young women who have ever been tested for HIV. The multivariable logistic regression analysis was used to assess the determinants of HIV testing at a 95% confidence interval (CI), and adjusted odds ratio (aORs) and p-values were reported. All analyses were adjusted using survey weights to account for unequal sampling probabilities.

**Results:**

The results showed that 31.4% (95% CI [29.63, 32.81]) of young women in Ghana had tested for HIV. The odds of HIV testing were likely to be higher among young women aged 20–24 (aOR = 2.24, 95% CI [1.75, 2.87]), those who were pregnant (aOR = 3.17, 95% CI [2.03, 4.95]) and those with one (aOR = 7.99, 95% CI [5.72, 11.17]), two (aOR = 10.43, 95% CI [6.47, 16.81]) or three or more children (aOR = 14.60, 95% CI [8.37, 25.48]) compared to their counterparts in the reference category. Women who had attained secondary education or higher (aOR = 2.66, 95% CI [1.67, 4.23]), were sexually active (aOR = 2.82, 95% CI [2.00, 3.97]), and in richer (aOR = 1.98, 95% CI [1.17, 3.34]) and richest wealth index (aOR = 1.99, 95% CI [1.10, 3.61]) were more likely to test for HIV than those with no formal education, who had not had sex before or in the poorest wealth index. Women from the Eastern (aOR = 1.69, 95% CI [1.04,2.72]) and Upper East regions (aOR = 2.62, 95% CI [1.44, 4.75]) were more likely than those in the Western region to get tested for HIV. However, the odds of testing for HIV were lower among women belonging to other religions (aOR = 0.43, 95% CI [0.23,0.82]) than Christians.

**Conclusion:**

The findings show that HIV testing is low among young women in Ghana. To address this issue, it is recommended that both government and non-governmental organizations collaborate to create effective programmes and strategies. These may include continuous health education, regular sensitization programs and making HIV testing services much more accessible and affordable, taking into consideration the sociodemographic characteristics of young women.

## Introduction

HIV/AIDS is one of the most critical public health and social problems in the world. It is still one of the leading causes of morbidity and mortality worldwide and costs the global economy and society billions of dollars every year in direct medical expenses and indirect socioeconomic costs [1]. Numerous people still contract the virus despite interventions and improvements in the understanding of HIV and its prevention. In 2016, it was estimated that 36.7 million individuals worldwide were living with HIV, and 1.8 million new HIV infections were recorded [2]. Recent estimates in the 2022 UNAIDs Global HIV & AIDS statistics—Fact sheet shows that this number had risen to 39 million, with 1.3 million new cases [3]. This shows that the virus is spreading quickly and that there are more people with the infection every day who have not been tested. Women between the ages of 15 and 24 are particularly at risk of contracting HIV, with approximately 26% of new cases reported worldwide [2]. A review of regional statistics reveals that the majority of HIV-positive individuals reside in low- and middle-income countries. West African women between the ages of 15 and 24 account for 22% of new HIV infections in the sub-Saharan African region, where 71% of new infections are identified [4]. In 2019, an estimated 26 million people in SSA were living with HIV [1].

HIV/AIDS in Ghana is characterized as mature, mixed, and generalized from an epidemiological perspective [5]. Recent studies have shown that the HIV prevalence rate among the general population in Ghana is 1.7%, affecting 334,713 people and accounting for over a thousand annual deaths, with the primary drivers being heterosexual contact and mother-to-child transmission [6]. However, there are variations in the prevalence rate among different groups and regions. Ghana’s urban areas have the highest frequency of HIV cases because of a variety of social and commercial contacts compared to rural areas. Ghana’s estimated HIV population in 2020 was 346,120, with females accounting for 66% and males comprising 34%, underscoring the heightened vulnerability of young women to the infection [7].

Young women are typically seen as being the most vulnerable to HIV/AIDS due to their increased engagement in risky sexual behaviors such as unprotected sex [8]. This is also a result of their biological make-up or physical structure. Due to the increased mucous area exposed during penile penetration, women are biologically more susceptible to infection than men [9]. Additionally, mother-to-child transmission during pregnancy and infancy exposes most young women to HIV [10]. In addition, lower cultural, social, and economic status in society make young women disproportionately at risk for HIV/AIDS. Women’s structural susceptibility to contracting HIV has been linked, among other things, to gender inequality [4].

The first and most crucial step in the fight against HIV/AIDS is perhaps knowing one’s HIV status. To this end, the Joint United Nations Programme on HIV/AIDS set the 95-95-95 target to be achieved by the end of 2025 [11]. By 2025, 95% of individuals living with HIV should be aware of their status, 95% of those diagnosed with the virus should be getting continuous antiretroviral therapy (ART), and 95% of those on such treatment should have viral suppression. Therefore, early diagnosis and early ART are crucial. The 95-95-95 target has proved successful in up scaling access to ART and knowledge on HIV-positive status. These efforts to increase testing and treatment emphasize the need for early diagnoses and ART. HIV testing is a crucial method of risk management and HIV/AIDS prevention. Expanding HIV testing coverage and education can considerably lower the number of new infections, especially among high-risk groups. Unfortunately, the few established HIV testing and prevention tools continue to face obstacles such as stigma, which hampers the desire for and uptake of HIV testing [12]. The limitation of not adhering to the HIV testing-care-treatment continuum is that it may have adverse effects on the global goal of achieving the 95-95-95 target [11].

Studies examining the uptake of HIV testing and its determinants among young women in Ghana are rare. An earlier study examined the relationship between ethnicity and HIV testing among adolescent girls and young women in Ghana and found that there was no association between ethnicity and HIV testing [5]. Given that young women are the most vulnerable, with a larger proportion of HIV cases recorded among them, it is important to understand the factors that influence their uptake of HIV testing. Therefore, in this study, we report the prevalence and factors associated with HIV testing among young women in Ghana. Findings of the present study highlight the HIV testing rate among young women and provide empirical information on the determinants worth considering in planning HIV eradication programs in Ghana.

## Methods

### Study setting

Ghana is situated on the West African coast, positioned between latitudes 4° North and 12° North and longitudes 4° West and 2° East, approximately 750 km north of the equator. According to the general report released by the Ghana Statistical Service (GSS) on the 2021 Population and Housing Census (PHC), Ghana has a population of about 30,832,019 [13]. In terms of demographics, the Ghanaian population has a significant proportion of young people. The 2010 Population and Housing Census indicated that young people aged 15–24 are approximately 4.9 million in number, representing 20% of the entire population of Ghana. HIV prevalence in Ghana is variable based on the geographic location, gender and age of the populations being examined.

### Data source

This study used data from the 2014 Ghana Demographic and Health Survey (GDHS). The GDHS is a nationally representative data that surveyed all 10 regions of Ghana. The Ghana Health Service (GHS) and GSS in collaboration with ICF International collected data on child health, family planning, malaria, health insurance, nutrition, HIV and AIDS, and maternal health as part of the GDHS. The GDHS also contains information on maternal mortality, gender, nutrition, awareness about HIV/AIDS, self-reported sexually transmitted infections (STIs), and other health issues relevant to the achievement of the Sustainable Development Goals (SDGs). A multistage sampling approach was adopted to sample respondents for the survey. The first phase of the sampling was characterized by the compilation of clusters encompassing enumeration areas (EAs) using those used for population and housing census. After this, households from each cluster were sampled, and inhabitants in these households were interviewed. The dataset is freely accessible at https://dhsprogram.com/data/available-datasets.cfm. Details of the DHS methodology have been reported elsewhere [14]. From the survey, a total of 9,396 women were interviewed. However, this study included 3,322 young women (15 to 24 years) who were asked if they had ever been tested for HIV and had complete data. The analysis excluded responses from participants with missing data. The GDHS final report provides detailed information on the method, pretesting, on-site staff training, sampling design, and selection. The manuscript has been drafted following guidelines outlined in the Strengthening the Reporting of Observational Studies in Epidemiology (STROBE) statement.

### Study variables

#### Dependent variable

The dependent variable for this study was HIV testing among young women. Reproductive age women included in the survey were asked “Have you ever been tested for HIV/AIDS?”. Information about the dependent variable was generated from this question. Respondents provided a “Yes” or “No” as a response to the question to indicate whether they had ever been tested for HIV or not. Based on their responses, a dichotomous response of Yes was coded ‘1’ when a young woman reported that she had been tested for HIV and ‘No’ was coded ‘0’ if she had not been tested.

#### Independent variables

Thirteen independent variables were included in this study and were selected based on their availability in the dataset and inferences from previous studies [10, 15–17]. The independent variables used in this study were currently pregnant, age of respondent, marital status, highest educational level, total children ever born, exposure to television, exposure to radio, exposure to newspaper, religion, ever had sex, wealth index, place of residence, and region.

### Statistical analysis

Stata version 14 was used to analyze the data. The study sample was described using descriptive analysis, weighted (v005/1,000,000) and checked for completeness. Descriptive, bivariate, and multivariable logistic regression analyses were performed on the data. First, descriptive analysis was performed for each variable, and the proportion of participants who had ever been tested for HIV was determined. The bivariate analysis was performed using the chi-square test to examine the associations between HIV testing and all the independent variables. All the variables, whether significant in the chi-square test or not were included in a multivariable analysis to determine their collective associations with HIV testing. The results obtained from the multivariable logistic regression analysis were presented as adjusted odds ratios (aORs) with 95% confidence intervals (CIs). The complex nature of the survey, was accounted for using the Stata command survey set (svy) taking into consideration weight, cluster and strata. In the analysis, *p*-values less than 0.05 were considered statistically significant.

### Ethical consideration

In this study, ethical clearance was not sought due to the public availability of the DHS dataset. The datasets were obtained from the MEASURE DHS after registration and approval were given for its usage. This was done through an application after registering the research project and describing how the data will be used as well as the analysis process. All ethical guidelines concerning the use of secondary datasets in the publication were strictly adhered to. Detailed information about the DHS data usage and ethical standards is available at http://goo.gl/ny8T6X.

## Results

### Sociodemographic characteristics of the participants

A total of 3,322 young women were included in this study. The sociodemographic characteristics of the study sample are shown in Table 1. A large number of the young women were not pregnant or unsure if they were pregnant (94.37%), and approximately half (50.14%) of them were aged 15–19 years. A higher proportion (75.40%) of the women had never being in union. A total of 73.53% had received a secondary/higher form of education. A total of 70.62% had never had a child. With exposure to a media source, 78.77% and 82.31% were exposed to television and radio, respectively, while only 26.17% were exposed to newspapers. The majority (79.82%) of the women were Christians. More than half (64.83%) had engaged in sexual intercourse. A higher proportion of the women were in the richer quintile of wealth, and 51.04% were urban residents. The majority of women were in the Greater Accra (17.91%) and Ashanti regions (18.54%).

### Bivariate association between HIV testing and the independent variables

Overall, only 1,043 (31.4%, 95% CI [29.63, 32.81]) out of the 3,322 participants indicated that they had been tested for HIV. From the bivariate analysis, current pregnancy status (*p* < 0.001), age of respondent (*p* < 0.001), marital status (*p* < 0.001), highest educational level (*p* = 0.013), total children ever born (*p* < 0.001), exposure to television (*p* = 0.037), exposure to radio (*p* = 0.008), religion (*p* = 0.041), ever had sex (*p* < 0.001), wealth index (*p* < 0.003) and region (*p* = 0.006) were significantly associated with HIV testing among young women (Table 1).

**Table 1:** Distribution of HIV testing across independent variables

Variable			Ever been tested for HIV		*P*-value
	Weighted N	Weighted %	No (%), 68.62	Yes (%), 31.38	
**Currently pregnant**					< 0.001
No/unsure	3135	94.37	70.43 [68.07,72.69]	29.57 [27.31,31.93]	
Yes	187	5.63	38.12 [30.57,46.28]	61.88 [53.72,69.43]	
**Age of respondent**					< 0.001
15–19	1666	50.14	86.18 [83.48,88.50]	13.82 [11.50,16.52]	
20–24	1656	49.86	50.95 [47.75,54.14]	49.05 [45.86,52.25]	
**Marital status**					< 0.001
Never married	2505	75.40	79.36 [77.03,81.52]	20.64 [18.48,22.97]	
Married	360	10.83	37.41 [31.87,43.29]	62.59 [56.71,68.13]	
Cohabiting	369	11.10	33.79 [27.76,40.39]	66.21 [59.61,72.24]	
Other	88	2.66	36.46 [24.97,49.73]	63.54 [50.27,75.03]	
**Educational level**					0.013
No formal education	269	8.09	64.25 [57.26,70.68]	35.75 [29.32,42.74]	
Primary	611	18.38	74.40 [69.79,78.52]	25.60 [21.48,30.21]	
Secondary+	2443	73.53	67.65 [64.79,70.39]	32.35 [29.61,35.21]	
**Parity**					< 0.001
0	2346	70.62	84.12 [82.14,85.92]	15.88 [14.08,17.86]	
1	593	17.84	33.26 [28.75,38.10]	66.74 [61.90,71.25]	
2	268	8.06	28.71 [22.69,35.58]	71.29 [64.42,77.31]	
3+	116	3.48	27.61 [19.72,37.18]	72.39 [62.82,80.28]	
**Exposed to television**					0.037
No	705	21.23	72.40 [68.58,75.91]	27.60 [24.09,31.42]	
Yes	2617	78.77	67.60 [64.77,70.30]	32.40 [29.70,35.23]	
**Exposed to radio**					0.008
No	588	17.69	75.52 [70.13,80.22]	24.48 [19.78,29.87]	
Yes	2734	82.31	67.13 [64.39,69.76]	32.87 [30.24,35.61]	
**Exposed to newspaper**					0.114
No	2453	73.83	67.67 [64.63,70.57]	32.33 [29.43,35.37]	
Yes	869	26.17	71.29 [67.80,74.54]	28.71 [25.46,32.20]	
**Religion**					0.042
Christianity	2652	79.82	67.27 [64.47,69.95]	32.73 [30.05,35.53]	
Islam	554	16.66	74.43 [69.16,79.06]	25.57 [20.94,30.84]	
Other	117	3.52	71.61 [60.28,80.74]	28.39 [19.26,39.72]	
**Ever had sex**					< 0.001
No	1168	35.17	92.85 [90.70,94.54]	7.15 [5.46,9.30]	
Yes	2154	64.83	55.47 [52.53,58.37]	44.53 [41.63,47.47]	
**Wealth index**					0.003
Poorest	582	17.53	76.54 [72.75,79.94]	23.46 [20.06,27.25]	
Poorer	633	19.05	68.44 [64.15,72.44]	31.56 [27.56,35.85]	
Middle	722	21.74	65.63 [61.51,69.54]	34.37 [30.46,38.49]	
Richer	740	22.26	64.23 [58.16,69.89]	35.77 [30.11,41.84]	
Richest	645	19.42	69.99 [65.09,74.48]	30.01 [25.52,34.91]	
**Place of residence**					0.533
Urban	1695	51.04	67.87 [64.29,71.26]	32.13 [28.74,35.71]	
Rural	1627	48.96	69.39 [66.05,72.53]	30.61 [27.47,33.95]	
**Region**					0.006
Western	397	11.94	70.65 [65.06,75.68]	29.35 [24.32,34.94]	
Central	312	9.39	61.07 [52.56,68.96]	38.93 [31.04,47.44]	
Greater Accra	595	17.91	69.42 [62.76,75.36]	30.58 [24.64,37.24]	
Volta	246	7.41	65.59 [58.89,71.72]	34.41 [28.28,41.11]	
Eastern	324	9.75	59.11 [54.00,64.02]	40.89 [35.98,46.00]	
Ashanti	616	18.54	71.31 [64.60,77.19]	28.69 [22.81,35.40]	
Brong Ahafo	306	9.22	67.12 [56.98,75.89]	32.88 [24.11,43.02]	
Northern	290	8.74	80.60 [72.91,86.52]	19.40 [13.48,27.09]	
Upper East	149	4.49	68.97 [61.78,75.34]	31.03 [24.66,38.22]	
Upper West	87	2.62	70.44 [64.57,75.7]	29.56 [24.3,35.43]	

Weighted N = Weighted Sample; Weighted %=Weighted Percentage

*p*-values generated from chi-square test.

### Multivariable analysis of factors associated with HIV testing

The results from the multivariable logistic regression analysis of the factors associated with HIV testing among young women in Ghana are displayed in Table 2. The results showed that women aged 20–24 (aOR = 2.24, 95% CI [1.75, 2.87]) have a higher odd of being tested for HIV than those below 20 years. The odds of being tested for HIV were higher among women who were pregnant (aOR = 3.17, 95% CI [2.03, 4.95]) than among those who were not pregnant. The odds of HIV testing increased in women with one (aOR = 7.99, 95% CI [5.72, 11.17]), two (aOR = 10.43, 95% CI [6.47, 16.81]) or three or more children (aOR = 14.60, 95% CI [8.37, 25.48]) than in those without any child. Women who had attained secondary education or higher (aOR = 2.66, 95% CI [1.67, 4.23]) had an increased odds of testing for HIV than those who had no formal education. The odds of testing for HIV increased in sexually active women (aOR = 2.82, 95% CI [2.00, 3.97]) compared to women who had never had sex. Women belonging to other religions had a decreased odds of testing for HIV compared to Christians (aOR = 0.43, 95% CI [0.23,0.82]). Women in the richer (aOR = 1.98, 95% CI [1.17, 3.34]) and richest (aOR = 1.99, 95% CI [1.10, 3.61]) wealth indices had higher odds of testing for HIV than those in the poorest wealth index. The odds of HIV testing was higher in women from the Eastern (aOR = 1.69, 95% CI [1.04,2.72]) and Upper East regions (aOR = 2.62, 95% CI [1.44, 4.75]) than in women from the Western region.

**Table 2:** Multivariable logistic regression of factors associated with HIV testing among young women in Ghana

Variable		*P* value
	aOR [95% CI]	
**Currently pregnant**		
No/unsure	1	
Yes	3.17 [2.03,4.95]	< 0.001
**Age of respondent**		
15–19	1	
20–24	2.24 [1.75,2.87]	< 0.001
**Marital status**		
Never married	1	
Married	1.45 [0.99,2.11]	0.054
Cohabiting	1.33 [0.93,1.91]	0.119
Other	1.50 [0.77,2.89]	0.230
**Educational level**		
No formal education	1	
Primary	1.23 [0.74,2.04]	0.430
Secondary+	2.66 [1.67,4.23]	< 0.001
**Parity**		
0	1	
1	7.99 [5.72,11.17]	< 0.001
2	10.43 [6.47,16.81]	< 0.001
3+	14.60 [8.37,25.48]	< 0.001
**Exposed to television**		
No	1	
Yes	1.09 [0.79,1.51]	0.591
**Exposed to radio**		
No	1	
Yes	1.28 [0.88,1.86]	0.194
**Exposed to newspaper**		
No	1	
Yes	1.30 [0.96,1.76]	0.090
**Religion**		
Christianity	1	
Islam	0.83 [0.58,1.18]	0.302
Other	0.43 [0.23,0.82]	0.010
**Ever had sex**		
No	1	
Yes	2.82 [2.00,3.97]	< 0.001
**Wealth index**		
Poorest	1	
Poorer	1.24 [0.80,1.92]	0.327
Middle	1.20 [0.75,1.91]	0.450
Richer	1.98 [1.17,3.34]	0.011
Richest	1.99 [1.10,3.61]	0.023
**Place of residence**		
Urban	1	
Rural	0.84 [0.60,1.18]	0.311
**Region**		
Western	1	
Central	1.49 [0.95,2.34]	0.081
Greater Accra	1.03 [0.65,1.63]	0.892
Volta	1.51 [0.84,2.71]	0.168
Eastern	1.69 [1.04,2.72]	0.032
Ashanti	1.20 [0.76,1.90]	0.441
Brong Ahafo	1.30 [0.72,2.35]	0.375
Northern	0.87 [0.42,1.77]	0.696
Upper East	2.62 [1.44,4.75]	0.002
Upper West	1.78 [0.91,3.50]	0.093

aOR adjusted odds ratio, CI confidence interval, 1 reference category

## Discussion

This study examined the prevalence of HIV testing among young women in Ghana and determined some crucial factors associated with the testing behaviors of these sampled population. The study unveiled a rather alarming situation, with only 31.4% of the study participants ever been tested for HIV. This finding hints at low HIV testing coverage among this population and highlights the need for targeted and timely intervention, as knowledge of one’s HIV serostatus helps to prevent and keep tabs on the spread of the infection. The HIV testing prevalence in this study, although concerning, surpasses rates elsewhere in the sub-Saharan African region [10, 18, 19] but is lower when compared to a significant number of studies conducted in a similar context [20–23]. A cross-national study carried out across a substantial portion of sub-Saharan African countries reported a pooled HIV testing prevalence of 64.4%, with Rwanda recording the highest proportion at 97.4% and the Democratic Republic of Congo the lowest at 20.2% [19]. The variations in HIV testing prevalence across countries can be ascribed to differing national strategies and policies employed to mitigate the spread of HIV [20].

Socioeconomic and demographic factors such as age, education level, region, religion, sexual activities, pregnancy status, total number of children ever born, and wealth index were identified as significant determinants impacting the likelihood of HIV testing among young women. Young women between the ages of 20 and 24 were more likely to ever test for HIV than their younger counterparts below age 20. This can be attributed to the perceived low risk of the latter age subgroup influenced by their relatively low sexual activity and limited awareness of potential HIV-related risks. A study conducted among young (15–24) women in eastern Africa reported a similar finding where the prevalence of HIV testing among young women increased with age [23].

A positive association between pregnancy status and HIV testing was observed, indicating that pregnant young women are more likely to take the HIV test. This result is a reflection of the global health directives that emphasize routine HIV testing during antenatal care in the quest to alleviate vertical transmission [24]. The results might also be attributed to the introduction of pregnancy-related point-of-care diagnostic tests, which include HIV testing in resource-constrained rural health facilities in Ghana [25].

Additionally, the study indicates that the prevalence of HIV testing was associated with the number of children ever born. Specifically, young women with at least three children had higher odds of testing for HIV than those with no children. This observation could be explained by the fact that having more children increases exposure and interaction with health care providers and services. Consequently, this heightened engagement holds the potential to not only elevate HIV awareness but also enhance proactive health-seeking behaviors.

With the attainment of a secondary education or higher, young women were more likely to test for HIV, unlike their peers with no formal education. This finding is consistent with studies conducted in other geographical settings [21, 23, 26]. This observation can be attributed to the enlightening effect of education on health, which enhances HIV awareness and empowers women to make well-informed healthcare choices, including the vital decision to test for HIV and ascertain their HIV status [10]. Well-educated women are likely to understand the need to test for their status and to know how to access testing services. It is therefore important to empower women academically and, more importantly, educate them on the need and means to test for their HIV status.

The study also identified financial status as a significant factor influencing HIV testing. Notably, young women with a higher wealth index had greater odds of undergoing HIV testing than those with a lower wealth index. In addition, even within the wealthy category, those designated as “richest” had a slightly higher odds ratio than their “richer” counterparts, further strengthening the association between wealth and HIV testing. An equivalent conclusion was drawn from studies conducted in Ethiopia [27] and Rwanda [28]. This finding hints at the presence of a potential financial hurdle to accessing healthcare services, including HIV testing in the country and calls for targeted interventions that will facilitate equitable healthcare services for economically disadvantaged populations. A positive correlation exists between an individual’s educational attainment and their wealth status. Individuals with higher education levels usually acquire specialized knowledge and expertise, which make them more eligible for well-paying professions.

Young women from the Eastern and Upper East regions were more likely than those in the Western region to get tested for HIV. This finding is in harmony with the outcome of a similar study carried out in the country [5]. With the Eastern region emerging as one of the regions with the highest regional HIV prevalence rate in Ghana, the findings from this study suggest the effect of risk perception on testing for HIV. This could explain why young women in the Eastern were more likely to test for HIV. Similar situation may exist in the Upper East region [29]. Additionally, health resource availability and access could also explain the observed geographical difference in HIV testing.

Christian young women were more likely to take an HIV test contrary to women from other religious backgrounds. This finding is consistent with a Nigerian study [30]. Religion can influence HIV prevention activities in various ways, including promoting HIV testing [31]. The findings from this study may be due to religious teachings on HIV and the establishment of social support groups in churches [32].

Consistent with other findings, the results of this study showed that sexually active young women were more likely to take HIV tests than those who were still in the realm of abstinence [18, 28]. This is probably because the former group is aware of the potential HIV-related risk associated with their behavior, and the prevailing notion that HIV as a typical sexually transmitted infection (STI) is predominantly transmitted through sexual means is widely known.

### Strengths and limitations

This study examines an important topic concerning HIV testing, utilizing DHS data to analyze the subject matter. The highly representative dataset was collected using standardized methods, significantly enhancing its reliability. However, there are some limitations to this study that must be considered when interpreting the results. First, as a cross-sectional design was employed, causation between determinants and HIV testing status cannot be established. Additionally, social desirability bias or cultural factors may influence participants’ responses to sensitive questions, potentially leading to inaccuracies in the data. The variable on lifetime HIV testing was self-reported and is thus prone to recall bias. Finally, the study was based on the 2014 GDHS. Although, this was the most recent DHS at the time this study was conducted, the findings may not reflect the current situation of HIV testing among young women in Ghana. Despite these limitations, the study’s findings can be valuable to the Ghana AIDS Commission, policymakers, and other stakeholders involved in HIV control in Ghana for the development of targeted interventions.

## Conclusion

This study highlights the low prevalence of HIV testing among young women in Ghana and the multifaceted factors that influence testing behaviors and patterns. Interventions and strategies aimed at increasing HIV testing among young women should be customized to suit their specific circumstances to yield positive outcomes. To address this issue, it is recommended that both government and non-governmental organizations collaborate to create effective programmes and strategies. These may include continuous health education, regular sensitization programs and making HIV testing services much more accessible and affordable, taking into consideration the sociodemographic characteristics of young women.

## Data Availability

The dataset supporting the conclusions of this article is available at https://dhsprogram.com/data/dataset/Ghana_Standard-DHS_2014.cfm?flag=0.
